# International Multi-Center Analysis of In-hospital Morbidity and Mortality of Low-Voltage Electrical Injuries

**DOI:** 10.3389/fmed.2020.590758

**Published:** 2020-11-11

**Authors:** Alexandra-Maria Warenits, Martin Aman, Clara Zanon, Felix Klimitz, Andreas A. Kammerlander, Anton Laggner, Johannes Horter, Ulrich Kneser, Anna Sophie Bergmeister-Berghoff, Klaus F. Schrögendorfer, Konstantin D. Bergmeister

**Affiliations:** ^1^Department of Emergency Medicine, Medical University of Vienna, Vienna, Austria; ^2^Center for Restoration of Extremity Function, Department of Surgery, Medical University of Vienna, Vienna, Austria; ^3^Department of Hand-, Plastic, and Reconstructive Surgery, Burn Center, University of Heidelberg, Heidelberg, Germany; ^4^Department of Hand- and Plastic Surgery, BG Trauma Center Ludwigshafen, University of Heidelberg, Heidelberg, Germany; ^5^Division of Cardiology, Department of Internal Medicine II, Medical University of Vienna, Vienna, Austria; ^6^Division of Oncology, Department of Internal Medicine I, Medical University of Vienna, Vienna, Austria; ^7^Department of Plastic, Aesthetic and Reconstructive Surgery, University Hospital St. Poelten, Karl Landsteiner University of Health Sciences, Krems, Austria; ^8^Department of Plastic, Aesthetic and Reconstructive Surgery, University Hospital St. Poelten, St. Poelten, Austria

**Keywords:** electrical injury, low-voltage, high-voltage, burn trauma, cardiac arrhythmia, neurological symptoms

## Abstract

**Background :** Patients with high- and low-voltage electrical injuries differ in their clinical presentation from minor symptoms to life-threatening conditions. For an adequate diagnosis and treatment strategy a multidisciplinary team is often needed, due to the heterogeneity of the clinical presentation. To minimize costs and medical resources, especially for patients with mild symptoms presenting after low-voltage electrical injuries, risk stratification for the development of further complications is needed.

**Methods :** During 2012–2019 two independent patient cohorts admitted with electrical injuries in two maximum care university hospitals in Germany and Austria were investigated to quantify risk factors for prolonged treatment, the need of surgery and death in low-voltage injuries. High-voltage injuries were used as reference in the analysis of the low-voltage electrical injury.

**Results :** We analyzed 239 admitted patients with low-voltage (75%; 276 ± 118 V), high-voltage (17%; 12.385 ± 28.896 V) or unclear voltage (8%). Overall mortality was 2% (*N* = 5) associated only with high-voltage injuries. Patients with low-voltage injuries presented with electrocution entry marks (63%), various neurological symptoms (31%), burn injuries (at least second degree) (23%), pain (27%), and cardiac symptoms (9%) including self-limiting thoracic pain and dysrhythmia without any therapeutic need. Seventy three percentage of patients with low-voltage injury were discharged within 24 h. The remaining patients stayed in the hospital (11 ± 10 days) for treatment of entry marks and burns, with an overall need for surgery of 12% in all low-voltage injuries.

**Conclusions :** The only identified risk factors for prolonged hospital stay in patients with low-voltage electrical injuries were the treatment of burns and electric marks. In this multi-center analysis of hospitalized patients, low-voltage electrical injuries were not associated with cardiac arrhythmia or mortality. Therefore, we suggest that asymptomatic patients, without preexisting conditions, with low-voltage injury can be discharged after an initial check-up without prolonged monitoring.

## Introduction

Electrical injuries occur frequently, with a broad range of symptoms and may lead to life-threatening secondary complications in certain risk constellations or high-risk patients (1, 2). Due to the manifold clinical presentations, including multiple organ systems, various disciplines are involved with often non-standardized diagnosis and treatment. Consequently, healthy patients may be overtreated, while others at risk suffer preventable secondary complications.

During electrical injuries, the body comes in contact with a source of electricity and the current is conducted through the body between entry and exit site. The tissue in-between is exposed to electricity and depending on exposure time, voltage and resistance potentially damaged. Therefore, certain electricity passages through the body are at higher risk to damage critical organs, most importantly the heart (3, 4). Generally, the voltage of the electric current and time of exposure is recognized as the main risk factor for the severity of injury. Subsequently, electrical injuries are categorized in low-voltage (<1,000 V) and high-voltage (>1,000 V) injuries. Most electrical injuries result from low-voltage current of 100–240 V, which is standard household electricity (5–8). Often, these patients present with no or mild symptoms, yet some are thought to be at risk for cardiac arrhythmia due to anecdotal reports of sudden cardiac death after electricity (4). To prevent late cardiac complications, a 24-h observation period under continuous cardiac surveillance has been suggested even for asymptomatic patients without preconditions (4, 9–12). Given the high number of short electrical contacts with low-voltage the costs of such a measure may unnecessarily divert important resources from other patients, since the actual risk has not been quantified (2, 13, 14).

Therefore, in this study we analyzed two independent patient cohorts of electrical injuries from Germany and Austria. We specifically investigate low-voltage injuries and use high-voltage injuries as a reference to quantify risk factors for death, prolonged treatment and need of surgery.

## Methods

### Study Design and Patient Analysis

We analyzed two independent patient cohorts from two maximum care University hospitals in Germany and Austria. Here, all patients treated for low-voltage electrical injuries were included, and high-voltage injuries used as reference. Records were obtained at the Medical University of Vienna in Austria for 2013–2019 and at the BG Trauma Center Ludwigshafen, Germany for 2012–2019. Patients were identified based on ICD-10 Codes (T75.0 Effects of lightning; T75.4 Effects of electric current; or W87.9 Exposure to unspecified electric current) or by free text search from the hospital's electronic records. We extracted data for gender, age, voltage, mortality, symptoms, ECG, blood sampling, need for surgery and hospital days. Blood sample parameters relevant for electrical injuries included serum creatine kinase (CK), creatine kinase-MB (CK-MB), myoglobin and troponin levels. Ethical approval was obtained from the local institutional review board at the Medical University of Vienna (EK Nr: 1575/2019) and for German collective from the data privacy institution (Landesärztekammer Rhld.-Pf., Mainz; EK Nr: 2020-15144).

### Data Management and Statistical Analysis

All data were reported anonymously to the study coordinator. Data and privacy management were according to the legislature of each country. All data analyses were conducted using Microsoft Excel in a two-stage manner by KDB, CZ, MA, and AB. Statistical analyses were conducted using SPSS Statistics Version 26 (IBM, USA). For descriptive statistics, the mean and the standard deviation were calculated for variables. Further statistical comparison between groups was conducted using either Student's *T*-Test, χ^2^ or Kruskal Wallis Test, see specifications in parentheses. A two-sided *p*-value < 0.05 was considered to indicate statistical significance.

## Results

### Patient Characteristics

**Figure 1:**
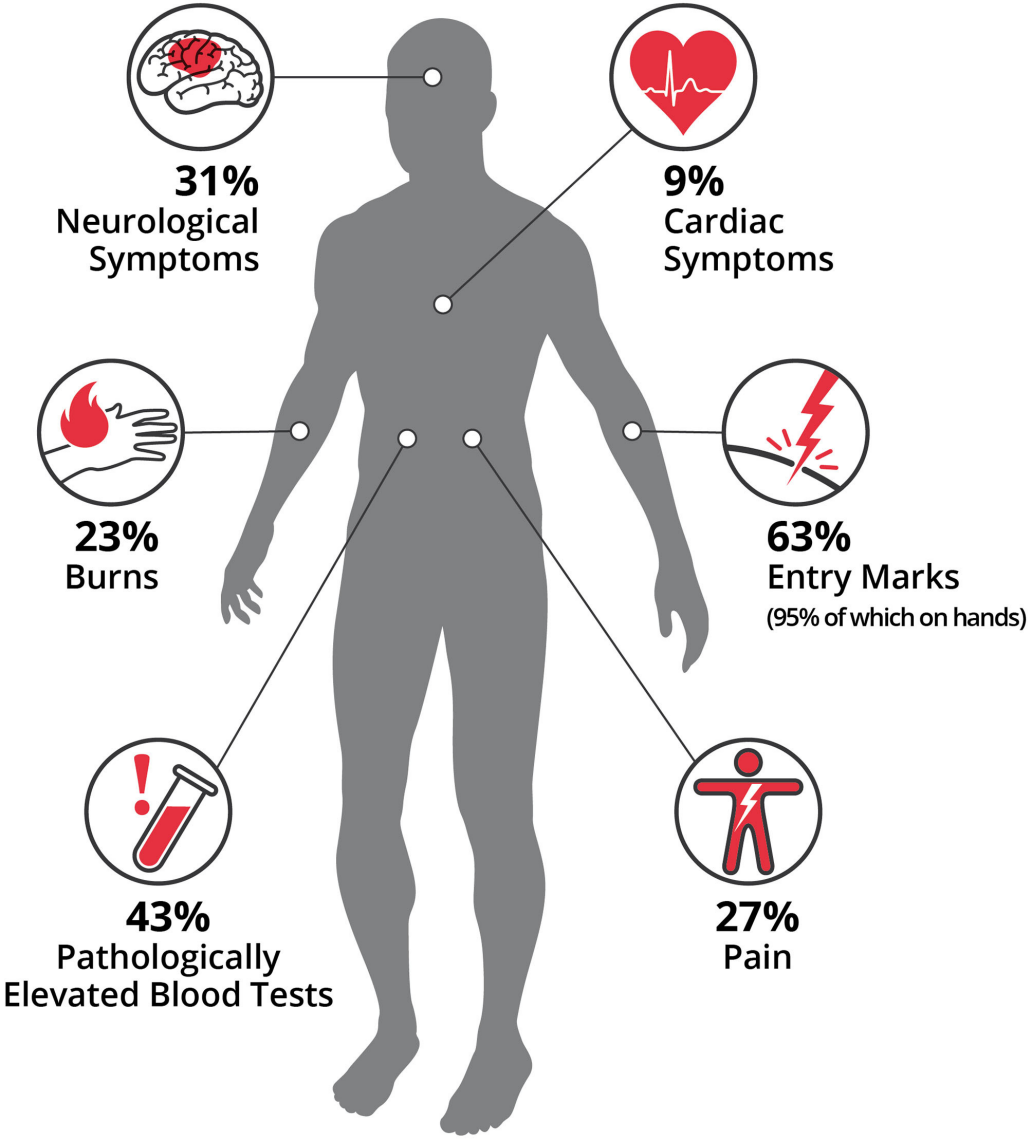
Patient characteristics. This study analyzed 239 patients of which 80% were male. The majority were work-related accidents (70%) and low-voltage injuries (75%). Overall, 43% were associated with 220–230 V the common household electricity in Europe. All fatalities were due to high-voltage current and none occurred in the low-voltage group. Patients had a median of two symptoms. Pathological ECGs were present in 14 and 17% thereof had pathological ECGs after 24 h. Of patients with low-voltage electric injuries, 27% required hospitalization beyond 24 h due to treatment of burns or entry marks.

A total of 239 patients were treated for electrical injuries in our patient cohort, of which 80% (*N* = 191) were male and 20% female (*N* = 47). Overall, average age was 33 ± 14 years, 33 ± 13 for male patients and 37 ± 14 for female patients. Ninety seven percentage (*N* = 230) were adults (>18a) and 94% of patients were between 18 and 65 years old, the standard age for work, with nine patients under 18 (14–17 years) and six above 70. The majority of electrical injuries were work-related (70%; *N* = 167), while 26% (*N* = 63) were non-work related and 4% (*N* = 9) not specified (Figure 1). The number of work accidents was higher in male patients (73%; *N* = 139) compared to females (60%; *N* = 28). Between centers, work accidents represented 55% of cases in Austria and 75% in Germany (*P* < *0.0001*). Pre-existing medical conditions were scarce and included most commonly arterial hypertension, coronary artery disease, diabetes.

### Voltage and Mortality

In 171 of 239 cases (72%), exact values for voltage associated with the injury was available. Overall, 75% were low-voltage injuries with an average of 276 ± 118 V (25–980 V), leading to 0% mortality. Conventional 220–230 V household electricity was responsible for 43% (*N* = 103/239) of injuries and mortality was 0%. In 17% the injury was caused by high-voltage with an average of 12.385 ± 28.896 V, leading to five deaths. All deaths were related to high-voltage. Overall mortality was 2% (*N* = 5) but 12% in high-voltage injuries (Figure 1). Thereof, one patient presented preclinically with ventricular fibrillation cardiac arrest and was transported with ongoing cardiopulmonary resuscitation to the hospital, where he died within hours despite the initiation of extracorporeal cardiopulmonary resuscitation. The other four patients died on the burn intensive care unit due to sepsis and/or multi-organ failure after 33 ± 28 days without primary failing of the heart. These patients had suffered burns of on average 44 ± 10% total body surface area.

In three of five deceased patients the exact causative voltage was unclear but could be determined as at least above 1,000 V. High-voltage injuries were significantly more likely (*P* < *0.027*) in work accidents (*N* = 23; 59%) compared to non-work accidents (*N* = 16; 41%).

### Symptoms in Low-Voltage Injuries

**Figure 2:**
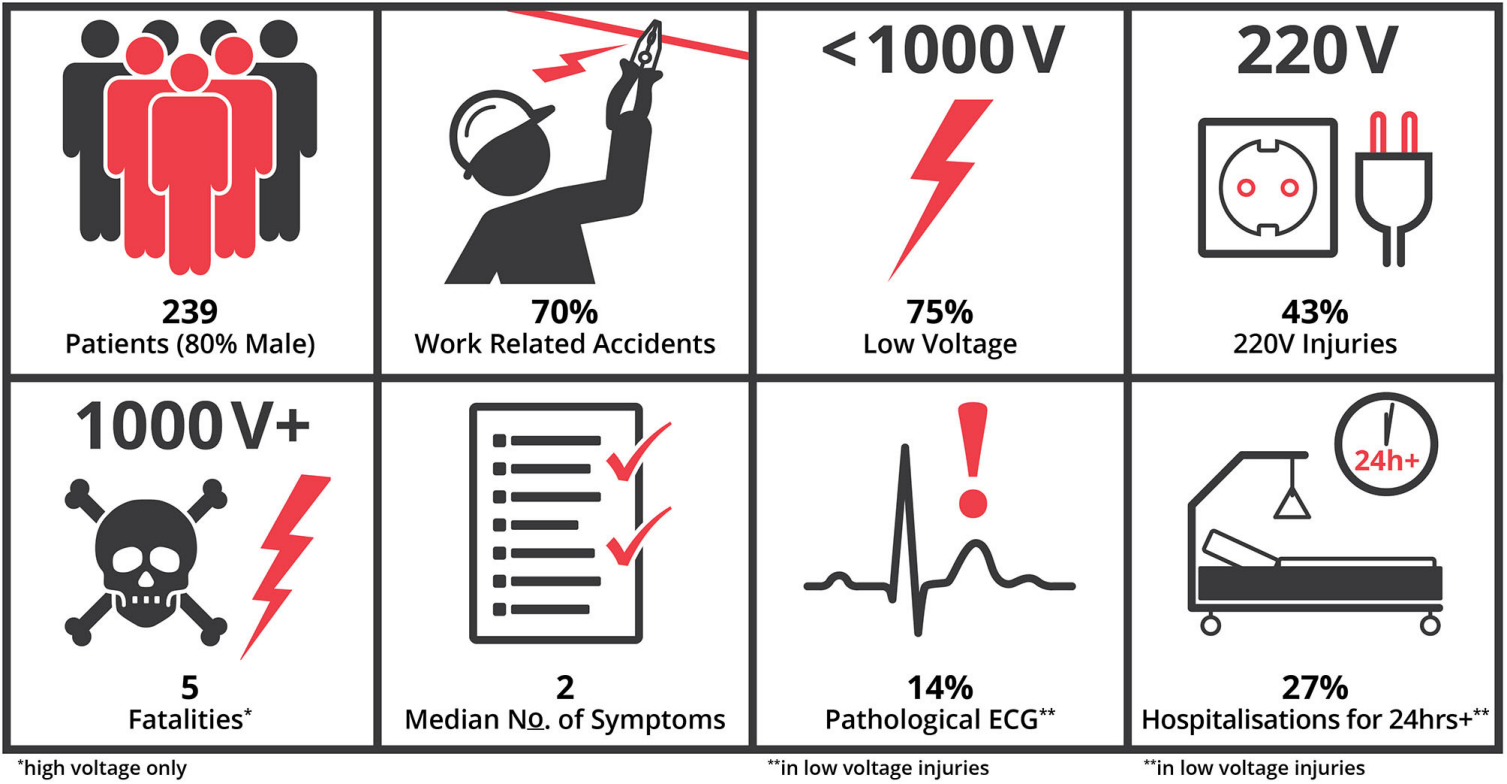
Symptoms in low-voltage electrical injuries. The median number of symptoms was two per patient. Patients suffered at first consultation from pain (27%). In this analysis, patients presented most dominantly with entry marks (63%), or pathologically elevated blood tests (43%), neurological symptoms (31%), and cardiac symptoms (9%). Patients presented (23%) at least with second degree burn injuries, most commonly (95%) at the hands. Only burns and electrocution marks were associated with the need of prolonged treatment beyond 24 h.

**Figure 3:**
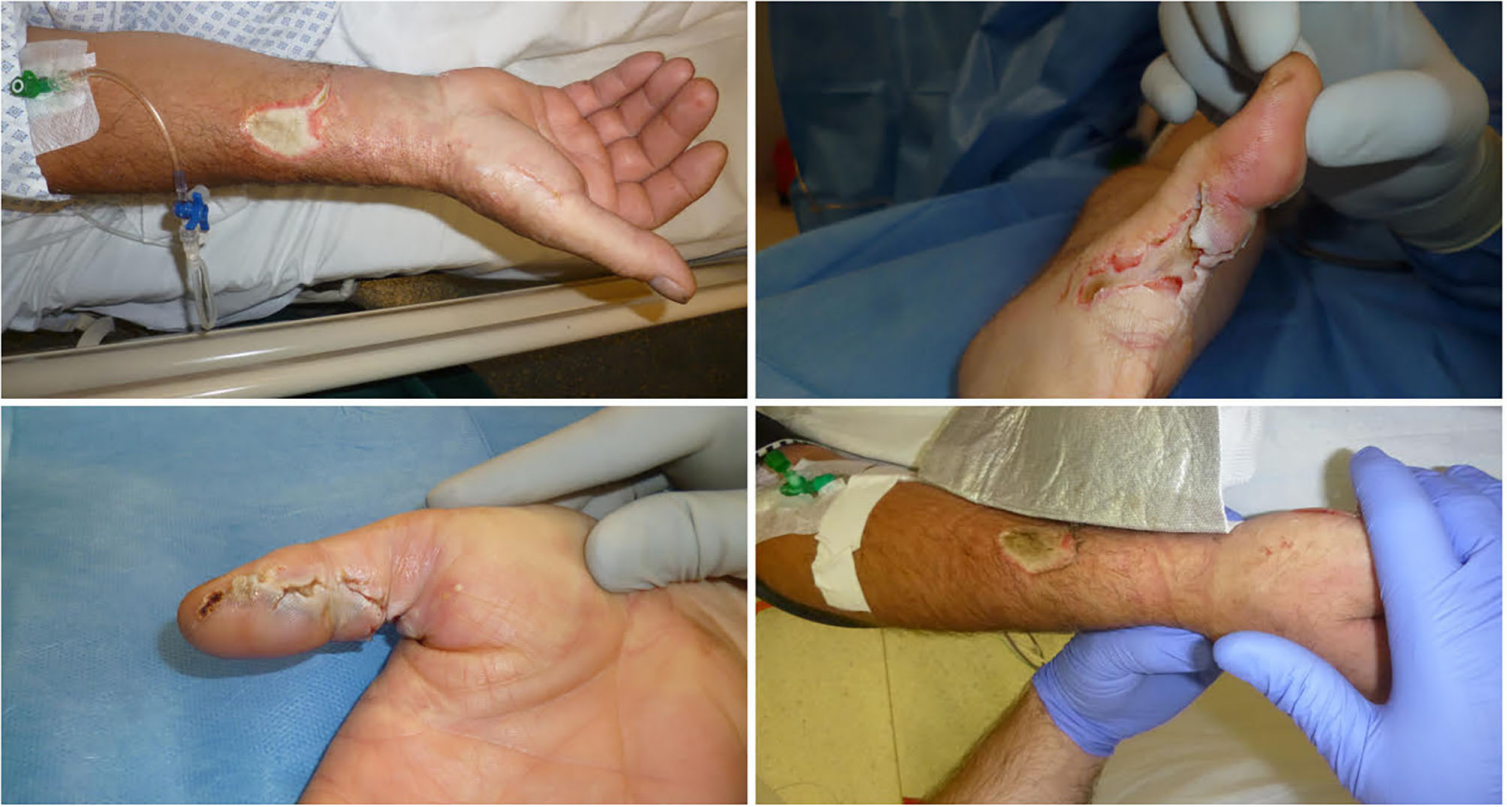
Low-Voltage entry wounds. Various presentations of entry marks of low voltage injuries, which show second to third degree burns. Here, surgical treatment and split skin grafts are often required for treatment. Such entry marks are generally believed to be a risk factor for secondary complications following electric injuries.

One hundred percentage of patients with high-voltage injuries presented symptoms at first contact in the hospital compared to 92% with low-voltage injuries. Median number of symptoms was two in low-voltage injuries, which were most commonly entry marks of the electrical contact (63%) or pathologically elevated blood tests (43%). In detail, elevated blood tests were troponin (high-sensitivity, T and I) (15%), myoglobin (24%), creatine-kinase (63%), and creatine-kinase MB (57%). Electric entry marks were most commonly at the hands (95%) (Figures 2, 3). Neurological symptoms were present in 31% of patients and included mainly temporary headache, dizziness at first consultation or persistent paresthesia at the electricity entry point, which were mainly the hands. In 23%, patients suffered at least second degree burn injuries, most commonly at the hands. Patients suffered from pain in 27% at first consultation (Figures 1–3).

### Cardiac Risk in Low-Voltage Injuries

Cardiac symptoms were present in 9% (*N* = 15/167) at first consultation in low-voltage injuries, no patient presented with cardiac arrest, or loss of consciousness at any time (Figure 2). These symptoms were mainly temporary tachycardia and thoracic pain, which usually resigned after few hours. ECG at first consultation was assessed in 98% (*N* = 233) of patients, except for five patients with low-voltage injuries, who declined the measure against the physician's advice. ECG abnormalities were present in 14% of low-voltage injuries (*N* = 24/167), of which 17% (*N* = 4) persisted after 24 h (Figure 1). Thereof, 55% had simultaneously elevated cardiac enzymes (CK, CK-MB, Troponin T/hsTroponin T/ Troponin I). On detailed inspection, these were either benign sinus bradycardia, extrasystoles or potentially pre-existing conditions (13%) such as non-specific T-wave abnormalities and peaked T wave (21%), right bundle branch block (21%), ventricular extra-beats (17%), sinus tachycardia (17%) or atrial fibrillation (4%). Two out of 90 asymptomatic patients (2%) developed ECG abnormalities, which were however both asymptomatic bradycardias with a frequency of 40–50 beats per minute in the young healthy patients and did not require any therapy. None of the patients in the entire low-voltage population required cardiac intervention or drug therapy. No patient was diagnosed with acute coronary syndrome.

### Duration of Stay and Need for Surgery in Low-Voltage Injuries

All Patients with risk factors yet without symptoms were discharged within 24 h except for one person that developed an unassociated diarrhea and was discharged after 48 h following temporary IV fluid treatment. Of all low-voltage patients, 73% were discharged within 24 h and the remaining 27% stayed for an average of 11 ± 10 days (Figure 2). Significant factors associated with longer duration than 24 h were entry marks (28% compared to 13% in below 24 h duration; *P* < *0.05*) and burns (63% compared to 10% in below 24 h duration; *P* < *0.001*). No other symptom or factor was significantly associated with need for hospital stay beyond 24 h. Overall need for surgery was 13% in all low-voltage injuries, with the majority (70%) being burn surgery or debridement under sedation and the rest simple wound treatments under local anesthesia due to concomitant injuries.

## Discussion

Our analysis presents a large cohort of adult patients with electrical injuries and did not show any mortality or immediate cardiac complication following low-voltage injuries. In contrast, 12% of the high-voltage electrical injuries suffered death as a complication of the accident. Comparable previous studies show similar results and together cannot confirm previous reports of late cardiac complications in asymptomatic adults (5–7) or children (8). Overall, these studies show a minimal mortality for low-voltage electrical injuries in patients treated in hospitals.

Historically, few anecdotal cases of initially asymptomatic patients who later suffered from cardiac complications have been reported in the literature (4, 15). However, a second analyses showed that only one patient had received an ECG at admission, and can therefore be confirmed as asymptomatic at first contact (11). The other patients, although presumably asymptomatic, may have already had ECG pathologies (12). Therefore, the actual risk for asymptomatic patients including normal ECGs with low-voltage electrical injuries is most likely smaller than previously thought (13, 14).

However, prolonged electrical contact which is known to cause immediate mortality is rare. This occurs for example during accidental submerging of electrical devices e.g., hairdryer in the shower or bathtub. These patients usually do not arrive at a hospital but often die at site of accident. Media reports in Germany and Austria indicate that both, especially accidental submerging of cellphones while bathing, are rare but increasing. In Austria approximately 300 electrical injuries (private and work) are officially registered per year (16). However, the actual number of low-voltage electrical injuries is supposedly much higher, as many are not reported or consult a doctor in the absence of symptoms. Thereby, results a presumably significant selection bias for in-hospital analyses, such as this one, as most healthy asymptomatic patients do not consult health services and are never registered. Therefore, the patients we see in hospitals are either driven by actual symptoms and the need for treatment or in the case of work accidents registration for the unlikely event of late problems and subsequent need for compensation. In our study, only 9% presented without any symptoms, of which only two patients have not suffered work accidents.

Overall, this suggests that the number of unreported low-voltage injuries is very high and thus the risk for asymptomatic patients even smaller than we can estimate from the currently published in-hospital data. In line, official mortality statistics indicate both high and low-voltage electrical injuries were responsible for 77 deaths in Germany, and two deaths in Austria in 2018 (16). This indicates that the risk for death is 89 deaths in an overall population of 91.856.920, equaling 0.097 deaths per 100.000 inhabitants. Unfortunately, there is no official distinction between low- and high-voltage injuries. However, the overall number is still very low.

Therefore, all published cohorts including the one presented in this study, show that low-voltage asymptomatic patients with normal ECG readings at first contact do not develop late cardiac complications (11, 17). Such cardiac complications have been described in only one confirmed case in many thousands in the literature, which had pathological ECG readings right at the first consultation (11). Based on the initial believe, that such late cardiac complications could occur in possibly any patient with electrical injury, some health care providers have suggested to monitor all patients for 24 h in the hospital (4, 9–12). Previous reports have already challenged the need for this measure in asymptomatic patients without risk factors (5–7, 17). In our study, ECG readings were performed at first contact and in case of risk factors or pathological ECGs after 24 h. Only two out of 90 asymptomatic patients developed pathological ECG readings, which were both benign bradycardias in young healthy and athletic patients. In contrast, 15% had initial pathological ECG readings, most of which were either preexisting conditions or benign arrhythmias and no patient required intervention or drug treatment. The only risk factors for hospital stay beyond 24 h were electrical marks or at least second-degree burns. Both are considered risk factors and require clinical surveillance for need of surgery and regular dressing changes. Both were also the only indicator for the need of surgery.

Electrical injuries in adults are common in the occupational setting and need due to their heterogeneity of organ systems involved specialists of multiple disciplines. Patients with low-voltage injuries often present with various symptoms to the emergency department. In times of overcrowding in worldwide emergency departments and limited resources like intermediate care units for a 24-h observational period, a risk stratification identifying potential complications in patients with low-voltage electrical injury is needed. Our data suggest that an in-patient stay with prolonged cardiac monitoring in healthy, asymptomatic low-voltage electrical injury patients after initial check-up is not necessary. This may help to prevent unnecessary monitoring of asymptomatic patients without preconditions, especially in time of COVID-19 and subsequent risk of infection.

This study is limited by the character of being a retrospective analysis. Future prospective analyses could reveal further risk stratifications.

Based on previous studies and our findings, we therefore suggest that asymptomatic low-voltage injuries patients with regular ECG readings do not require prolonged surveillance and repeated ECG readings (5, 13, 14, 17, 18). In line, the European Resuscitation Council states that only patients with the following risk factors should be monitored in hospital: (1) history of cardiorespiratory problems, (2) loss of consciousness, (3) cardiac arrest, (4) electrocardiographic abnormalities, or (5) soft-tissue damage and burns (1). In our study, even low-voltage injuries with risk factors did not suffer from late consequences. Other large studies report similar results and, therefore, the overall risk seems also low in the population at risk (1, 7, 19). However, contrary to the ERC guidelines, we suggest that patients with high-voltage injuries should be monitored, given the significantly higher mortality rate here and in other studies (10, 20). Although, the distinction between low- and high-voltage injuries has been artificially made at 1,000 V, in this and other studies patients were more likely to die above 1,000 V contact. Autopsy data of patients who died following electricity, suggests that high-voltage injuries have a high risk of death and most victims died at 2,400 V and above (10). Therefore, due to mortality rates of up to 30% and higher cardiac arrythmias rates (21), it seems necessary to monitor these patients even in the absence of symptoms and confirm the patients' well-being. Unfortunately, the currently available data does not allow the risk stratification of high-voltage injury patients which had exactly 1,000 V contact or slightly above. Given the significantly higher risk of mortality, the often unclear causative voltage and the fact, that many high-voltage patients have risk factors (e.g., burns or electrical marks), it seems a necessary precaution to aid the many different specialties involved in electrical injuries. Hereby, diagnosis and treatment could be standardized until more evidence is available to specify the needs of these patients.

## Conclusion

In this international two-center analysis of adult low-voltage electrical injuries, we did not find any associated cardiac arrythmias or mortality. We specifically analyzed patient morbidity and found electrical marks most commonly. The treatment of burns and electrical marks were the only factors statistically responsible for prolonged hospital stays beyond 24 h.

We recommend, that even patients with low-voltage injuries, without history of loss of consciousness, arrhythmias or initial cardiac arrest should receive an initial 12-lead ECG, physical examination and measurement of cardiac enzymes, including high-sensitivity cardiac troponin to asses any myocardial injury. In case of any pathology (e.g., elevated biomarkers, chest pain, ECG abnormalities) health professionals should follow the standardized cardiovascular guidelines for diagnosing and treatment of patients without presenting with an electrical injury. Our analyses suggest that asymptomatic low- voltage electrical injuries without preexisting conditions can be dismissed after initial check-up without prolonged monitoring.

## Data Availability Statement

The data analyzed in this study was subject to the following licenses/restrictions: The datasets used and/or analyzed during the current study are available from the corresponding author or the first authors on reasonable request. Requests to access these datasets should be directed to Konstantin D. Bergmeister, kbergmeister@gmail.com.

## Author Contributions

KB, A-MW, MA, and CZ designed the concept. All authors analyzed data, contributed their specific expertise, wrote the manuscript, and revised it critically and approved the final version of the manuscript.

## Conflict of Interest

The authors declare that the research was conducted in the absence of any commercial or financial relationships that could be construed as a potential conflict of interest.

## References

[1] Truhlár A Deakin CD Soar J Khalifa GE Alfonzo A Bierens JJ. European resuscitation council guidelines for resuscitation 2015: Section 4. Cardiac arrest in special circumstances. Resuscitation. (2015) 95:148–201. 10.1016/j.resuscitation.2015.07.017 26477412

[2] Zemaitis MR Foris LA Lopez RA Huecker MR. Electrical Injuries. Treasure Island (FL): StatPearls (2020). 28846317

[3] Geddes LA Bourland JD Ford G. The mechanism underlying sudden death from electric shock. Med Instrum. (1986) 20:303–15. 3543629

[4] Jensen PJ Thomsen PE Bagger JP Norgaard A Baandrup U. Electrical injury causing ventricular arrhythmias. Br Heart J. (1987) 57:279–83. 10.1136/hrt.57.3.279 3566986 PMC1216425

[5] Arrowsmith J Usgaocar RP Dickson WA. Electrical injury and the frequency of cardiac complications. Burns. (1997) 23:576–8. 10.1016/S0305-4179(97)00050-8 9568327

[6] Pilecky D Vamos M Bogyi P Muk B Stauder D Racz H. Risk of cardiac arrhythmias after electrical accident: a single-center study of 480 patients. Clin Res Cardiol. (2019) 108:901–8. 10.1007/s00392-019-01420-2 30771067 PMC6652167

[7] Bailey B Gaudreault P Thivierge RL. Cardiac monitoring of high-risk patients after an electrical injury: a prospective multicentre study. Emerg Med J. (2007) 24:348–52. 10.1136/emj.2006.044677 17452703 PMC2658483

[8] Bailey B Gaudreault P Thivierge RL Turgeon JP. Cardiac monitoring of children with household electrical injuries. Ann Emerg Med. (1995) 25:612–7. 10.1016/S0196-0644(95)70173-7 7741337

[9] Leibovici D Shemer J Shapira SC. Electrical injuries: current concepts. Injury. (1995) 26:623–7. 10.1016/0020-1383(95)00130-2 8550171

[10] Bailey B Forget S Gaudreault P. Prevalence of potential risk factors in victims of electrocution. Forensic Sci Int. (2001) 123:58–62. 10.1016/S0379-0738(01)00525-4 11731198

[11] Fatovich DM. Delayed lethal arrhythmia after an electrical injury. Emerg Med J. (2007) 24:743. 10.1136/emj.2007.050245 17901293 PMC2658458

[12] Sharma BC Patial RK Pal LS Saunkhla J Thakur SS. Electrocardiographic manifestations following household electric current injury. J Assoc Physicians India. (1990) 38:938–9. 2096133

[13] Kramer C Pfister R Boekels T Michels G. Cardiac monitoring always required after electrical injuries? Med Klin Intensivmed Notfmed. (2016) 111:708–14. 10.1007/s00063-015-0107-y 26496987

[14] Searle J Slagman A Maass W Mockel M. Cardiac monitoring in patients with electrical injuries. An analysis of 268 patients at the Charite Hospital. Dtsch Arztebl Int. (2013) 110:847–53. 10.3238/arztebl.2013.0847 24399026 PMC3888927

[15] Kose S Iyisoy A Kursaklioglu H Demirtas E. Electrical injury as a possible cause of sick sinus syndrome. J Korean Med Sci. (2003) 18:114–5. 10.3346/jkms.2003.18.1.114 12589099 PMC3054994

[16] Privatstiftung EG Elektrounfaelle in Oesterreich (1999–2018). Available online at: https://www.esf-vienna.at/de/elektrounfaelle-oesterreich-1999-2018 (accessed January 14, 2020).

[17] Fatovich DM Lee KY. Household electric shocks: who should be monitored? Med J Aust. (1991) 155:301–3. 10.5694/j.1326-5377.1991.tb142285.x 1895971

[18] Blackwell N Hayllar J. A three year prospective audit of 212 presentations to the emergency department after electrical injury with a management protocol. Postgrad Med J. (2002) 78:283–5. 10.1136/pmj.78.919.283 12151571 PMC1742340

[19] Fish RM. Electric injury, part III: cardiac monitoring indications, the pregnant patient, and lightning. J Emerg Med. (2000) 18:181–7. 10.1016/S0736-4679(99)00190-0 10699519

[20] Arnoldo BD Purdue GF Kowalske K Helm PA Burris A Hunt JL. Electrical injuries: a 20-year review. J Burn Care Rehabil. (2004) 25:479–84. 10.1097/01.BCR.0000144536.22284.5C 15534455

[21] Alexander Beck GK Mark Bischoff Stromunfall und Verbrennung. Notfallmedizin up2date. (2008) 3:25–40. 10.1055/s-2007-989449

